# Risk of Recurrence of Hepatocarcinoma after Liver Transplantation: Performance of Recurrence Predictive Models in a Cohort of Transplant Patients

**DOI:** 10.3390/jcm12175457

**Published:** 2023-08-23

**Authors:** Antonio Cuadrado, José Ignacio Fortea, Carlos Rodríguez-Lope, Ángela Puente, Vanesa Fernández-Vilchez, Victor Jose Echavarria, Federico José Castillo Suescun, Roberto Fernández, Juan Andrés Echeverri, Mar Achalandabaso, Enrique Toledo, Raúl Pellón, Juan Carlos Rodríguez Sanjuan, Javier Crespo, Emilio Fábrega

**Affiliations:** 1 Gastroenterology and Hepatology Department, Clinical and Translational Research in Digestive Diseases, Valdecilla Research Institute (IDIVAL), Marqués de Valdecilla University Hospital, 39011 Santander, Spain; jifortea@gmail.com (J.I.F.); carlos.rodriguezdelope@scsalud.es (C.R.-L.); angelam.puente@scsalud.es (Á.P.); javiercrespo1991@gmail.com (J.C.); emilio.fabrega@scsalud.es (E.F.); 2 General Surgery Service, Marqués de Valdecilla University Hospital, 39008 Santander, Spain; federicojose.castillo@scsalud.es (F.J.C.S.); roberto.fernandez@scsalud.es (R.F.); juanandres.echeverri@scsalud.es (J.A.E.); mariadelmar.achalandabaso@scsalud.es (M.A.); enrique.toledo@scsalud.es (E.T.); juancarlos.rodriguezs@scsalud.es (J.C.R.S.); 3 Radiology Service, Marqués de Valdecilla University Hospital, 39008 Santander, Spain; raul.pellon@scsalud.es

**Keywords:** liver transplantation, hepatocellular carcinoma, tumor recurrence, Milan criteria, pre-MORAL risk, RETREAT, obesity, body mass index

## Abstract

Liver transplantation (LT) is a curative treatment for early-stage hepatocellular carcinoma (HCC) unsuitable for surgical resection. However, tumor recurrence (TR) rates range from 8% to 20% despite strict selection criteria. The validation of new prognostic tools, such as pre-MORAL or RETREAT risks, is necessary to improve recurrence prediction. A retrospective study was conducted at Marqués de Valdecilla University Hospital in Cantabria, Spain, between 2010 and 2019 to determine the rate of TR in LT patients and identify associated factors. Patients with liver-kidney transplantation, re-transplantation, HIV infection, survival less than 90 days, or incidental HCC were excluded. Data on demographic, liver disease-related, LT, and tumor-related variables, as well as follow-up records, including TR and death, were collected. TR was analyzed using the Log-Rank test, and a multivariate Cox regression analysis was performed. The study was approved by the IRB of Cantabria. TR occurred in 13.6% of LT patients (95% CI = 7.3–23.9), primarily as extrahepatic recurrence (67%) within the first 5 years (75%). Increased TR was significantly associated with higher Body Mass Index (BMI) (HR = 1.3 [95% CI = 1.1–1.5]), vascular micro-invasion (HR = 8.8 [1.6–48.0]), and medium (HR = 20.4 [3.0–140.4]) and high pre-MORAL risk (HR = 30.2 [1.6–568.6]). TR also showed a significant correlation with increased mortality. Conclusions: LT for HCC results in a 13.6% rate of tumor recurrence. Factors such as BMI, vascular micro-invasion, and medium/high pre-MORAL risk are strongly associated with TR following LT.

## 1. Introduction

Hepatocellular carcinoma (HCC) is the most common primary liver malignancy, accounting for approximately 75–85% of all primary liver cancers worldwide [1,2]. Liver transplantation (LT) is considered the best curative treatment option for patients with early-stage HCC (within the Milan criteria), with 5-year survival rates of approximately 70% [3]. Despite the use of restrictive criteria, recurrence is still high, affecting between 8% and 20% of cases, and is a significant predictor of survival after LT [4]. Tumor recurrence (TR) occurs in 75% of cases during the first 2 years after LT (early recurrence) [3,4,5].

Several factors have been identified as predictors of HCC recurrence after LT. Preoperative tumor characteristics, such as tumor size, number and vascular invasion are, on the basis of the Milan criteria, successfully introduced by Mazzafero to predict the risk of recurrence and, consequently, the suitability of liver transplantation [6]. Other pre-transplantation risk factors include serum biomarkers, such as alpha-fetoprotein (AFP) levels and the neutrophil to lymphocyte ratio (NLR), obesity, bridging therapy or time to transplantation [7,8,9]. Tumor differentiation and microvascular invasion are two well-known predictors of prognosis only available in a morphological examination of the liver explant [10,11]. Additionally, factors related to the LT procedure, such as immunosuppressive therapy and delayed time to transplantation, have also been identified as predictors of HCC recurrence [5,9]. Taking advantage of this knowledge, there have been attempts at creating models and scales of risk of TR based on pathomorphological, biochemical and clinical parameters being the “Moral” and RETREAT scales the most commonly used [12,13]. Both scores, which incorporate different predictor variables, such as AFP, NLR, tumor number and size, tumor differentiation and microvascular invasion, have shown to be superior in predicting the risk of recurrence than the Milan criteria [9,13]. Other models also incorporate variables such as cancer etiology (hepatitis C virus “HCV” infection or another) [14], MELD classification extended by the sodium level (Model for End-Stage Liver Disease-sodium- MELD-Na), cirrhosis etiology or response to locoregional therapy [15,16]. However, the validity and suitability of each of these variables and/or models in a given transplant unit or geographical setting may vary.

The aim of this study is to estimate the rate of tumor recurrence in patients transplanted for HCC and identify relevant factors contributing to recurrence in a tertiary hospital in northern Spain. Furthermore, we sought to externally validate the predictive recurrence models Moral and RETREAT by assessing their predictive capacity within our cohort. 

## 2. Materials and Methods

### 2.1. Design of the Study and Population

We conducted an observational retrospective study in the Marques de Valdecilla University Hospital (Cantabria, Spain), an academic tertiary care center. All patients aged ≥18 years who received a liver transplant (LT) in our center between January 2010 and December 2019 due to an HCC were selected to participate. Exclusion criteria were liver-kidney transplantation, retransplant, acute liver failure, preoperative sepsis, HIV infection, death during the first 90 days post-transplant and incidental HCC in the explant (mainly because some pre-transplant variables, which were assessed as predictors of TR, were not available in this particular case). Electronic medical records were recorded as demographic and anthropometric variables, vascular risk factors including smoking habits, etiology of the liver disease and other pre-transplant hematologic and biochemical variables, such as AFP (both at diagnosis and at the closest assay before LT), neutrophil, lymphocytic and platelet counts and renal function as well as the MELD score. The following LT-related variables were also considered: age, CMV state and type of donor (brain vs. circulatory deceased donor), ischemic time, CMV recipient state, CMV infection and disease, rejection and need of steroid bolus, immunosuppressive regimen at the end of the first year, body mass index (BMI), tumor-related variables (nodule size and number both at radiological diagnosis, at the closest assessment before LT and also in the explanted liver, histological grade, microvascular invasion in the explanted liver and bridging therapy). Finally, follow-up events such as TR, time to TR and death were also recorded. Predictor models of recurrence (pre-Moral, post-Moral, Combo-Moral and RETREAT) were appropriately constructed as previously described [12,13].

### 2.2. Statistical Analysis

The descriptive analysis involved estimating proportions for discrete variables and means with their 95% CIs or medians with interquartile ranges for continuous variables. In the univariable analysis, comparisons between groups for categorical variables were conducted using the chi-square test or Fisher’s exact test. For continuous variables, either the independent samples t-test was used for normally distributed data or non-parametric tests (Mann–Whitney U test or median test) were employed when normality assumptions were not met. The normality assumption was assessed using the Kolmogorov-Smirnov test or Shapiro–Wilk test. Survival analysis was performed using the Kaplan-Meier method to assess the probability and risk of TR. Differences in survival curves were then evaluated using the log-rank test. Additionally, we conducted Cox regression analysis to estimate hazard ratios and 95% confidence intervals for the predictors of TR. The forward conditional method was used, introducing related variables in univariable analysis (*p* < 0.1). Statistical significance was considered for values of *p* < 0.05. The statistical analysis was performed using IBM SPSS Statistics v22.0 for Mac (IBM Corp., Armonk, NY, USA).

### 2.3. Ethics 

The study protocol conformed to the ethical guidelines of the 1975 Declaration of Helsinki as reflected in a priori approval by the Ethics Committee for Clinical Research of Cantabria (act 3/2023). A waiver of informed consent was provided since the study was considered a retrospective review.

## 3. Results

### 3.1. Study Population

**Figure 1:**
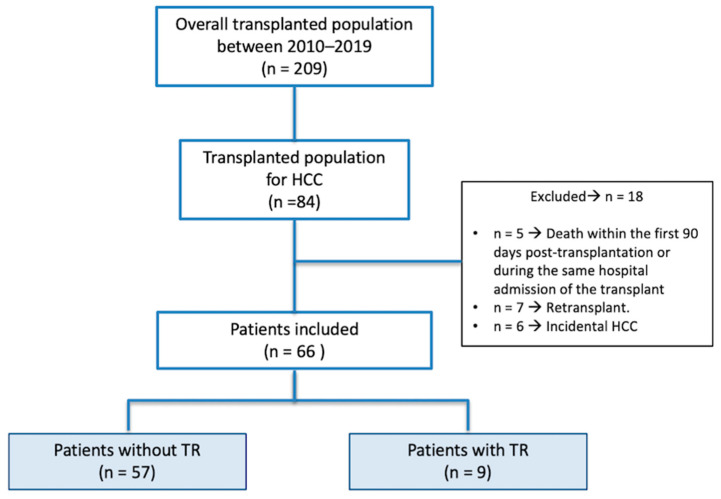
Flowchart of the study.

Between 2010 and 2019, a total of 209 liver transplants were performed, with 84 of them indicated for HCC (Figure 1). Eighteen cases were excluded from the analysis due to meeting specific exclusion criteria. Subsequently, data from 66 transplanted patients were analyzed. Most of the patients were males (n = 58; 87.9%) with a median age at transplantation of 60 years (interquartile range, IQR = 53.5–63.5). The median follow-up time for the patients in our cohort was 73.9 months (IQR = 45.1–97.3).

The most frequent etiologies of liver disease were alcohol-related (34.8%) and HCV infection (31.8%) (Table 1). Patients were listed for transplantation with a median MELD score of 10 (IQR: 8–12) and spent a median of 4 months on the waiting list (IQR: 2–8). The median time from HCC diagnosis to transplantation was 9 months (5.0–15.5). The incidence rates of CMV infection, histologically confirmed rejection and the requirement for steroid bolus in cases of confirmed or suspected rejection are presented in Table 1. At the end of the first year, most patients (68.2%) received calcineurin inhibitors (CNIs) as monotherapy. A small group (9.1%) were on monotherapy with an mTOR inhibitor (everolimus). TR patients received mTOR monotherapy more frequently, while non-TR patients were predominantly treated with CNI, either as monotherapy or in combination (*p* = 0.029) (Table 1).

### 3.2. Tumor Characteristics (Pre-Transplant and Explant)

Sixty-five patients (98.5%) met the Milan criteria at diagnosis. The mean number of nodules at diagnosis was 1.7 (95% CI = 1.5–1.9), with the mean size of the largest nodule being 29.1 mm (26.7–31.6). The size of the largest nodule was significantly larger in the group with recurrence (35.8 mm; 95% CI = 28.6–42.9) compared to the non-recurrence group (28.1 mm [25.5–30.7]; *p* = 0.028) (Table 1). No patient underwent downstaging (the only patient who was included despite exceeding the Milan criteria was a 31-year-old patient with three nodules, the largest of which measured 50 mm at diagnosis and was transplanted within the first month on the waiting list). The majority of patients received bridge therapy before transplantation (n = 51; 77.3%), with radiofrequency/microwave ablation being the most common treatment (56.1% of patients). The median AFP at waiting list inclusion was 4.3 ng/mL (IQR = 2.4–11.9), with a slightly lower value at the closest assessment prior to transplantation (4.2; 2.4–10.4).

The number and size of nodules in the explant are summarized in Table 1. No significant differences were observed between individuals who experienced recurrence and those who did not (Table 1).

### 3.3. Tumor Recurrence

**Figure 2:**
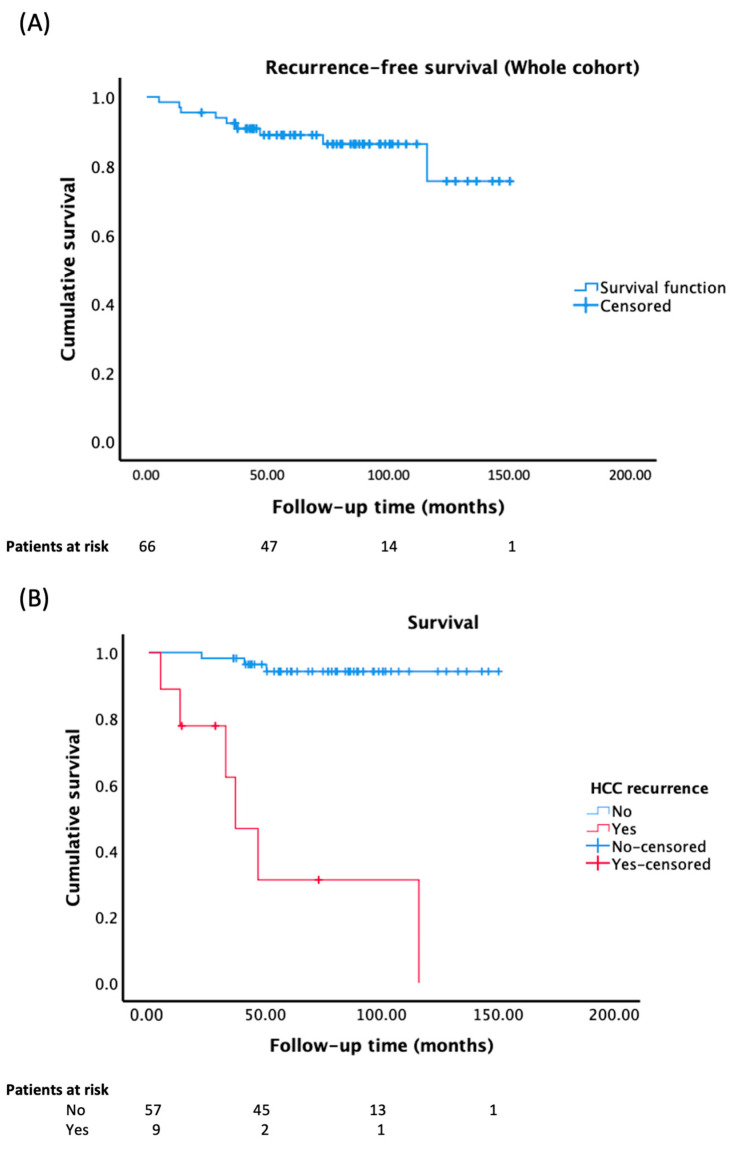
(**A**) Recurrence-free survival in the overall cohort; (**B**) survival curves based on tumor recurrence status (Log-rank (Mantel-Cox): *p* < 0.001).

Nine patients (13.6%; 95% CI = 7.3–23.9) experienced HCC recurrence. In six patients (66.7%), tumor recurrence occurred at an extrahepatic level, with some cases involving multiple locations at the time of diagnosis: bone (3), lung (2), adrenal gland (2), and other locations (2). The remaining three patients experienced intrahepatic recurrence. Most recurrences occurred early: 77.8% within five years, 55.6% within three years, and 33.3% within two years post-transplantation. The mean recurrence-free survival time in the overall cohort was 130.91 months (95% CI: 118.9–142.9). The probability of tumor recurrence at one, three, and five years was 5%, 11%, and 11%, respectively, in the overall cohort (Figure 2A).

### 3.4. Factors Associated with Tumor Recurrence

In the univariable analysis, TR was associated with the size of the largest nodules at diagnosis, BMI, obesity and the presence of vascular micro-invasion (*p* = 0.036, *p* = 0.037, 0.039 and *p* = 0.040, respectively; Table 2). Furthermore, the composite predictive models, pre-Moral, Combo-Moral and RETREAT risk scores demonstrated a significant association with an increased risk of TR, whereas the post-Moral model did not show such significant associations (Table 2). Pre-transplant AFP demonstrated a non-significant association trend (Table 2) 

In the multivariable analysis using Cox regression, BMI (Hazard Ratio [HR] = 1.3 [1.1–1.5]; *p* = 0.006), vascular micro-invasion (HR = 8.8 [1.6–48.0]; *p* = 0.012) and medium and high pre-MORAL risk (HR = 20.4 [3.0–140.4]; *p* = 0.002 and HR = 30.2 [1.6–568.6]; *p* = 0.023 respectively) remained associated with the risk of TR (Table 2). When obesity (defined as BMI ≥ 30) was introduced in the multivariate model instead of BMI, it was also found to have a significant association with the risk of TR (HR = 8.6, 95% CI [1.8–42.1]; *p* = 0.008). Furthermore, vascular micro-invasion and pre-Moral risk continued to show significant associations with the risk of TR, as indicated in Table 2.

**Figure 3:**
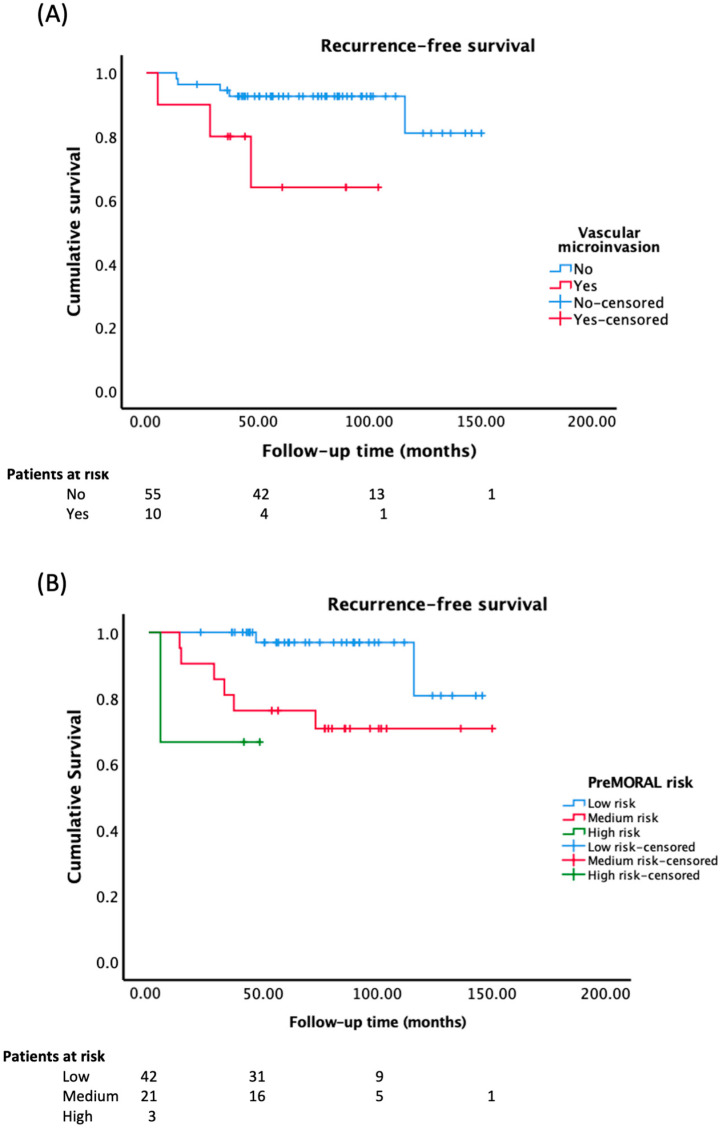
(**A**) Recurrence-free survival based on the presence of vascular micro-invasion in the graft (Log-rank (Mantel-Cox): *p* = 0.023). (**B**) Recurrence-free survival according to the pre-MORAL risk. (Log-rank (Mantel-Cox): *p* = 0.012).

In patients with vascular micro-invasion, the mean recurrence-free survival was 77.5 months (CI 95% = 52.6–102.4), compared to 137.1 months (125.9–148.3) in those without micro-invasion (Log-rank (Mantel-Cox): *p* = 0.023) (Figure 3A).

The cumulative probability of tumor recurrence at 1 year, 3 years, and 5 years for patients with microinvasion was 10%, 32%, and 32%, respectively, while it was 4%, 8%, and 8% in those without micro-invasion. In patients with low pre-MORAL risk, the mean recurrence-free survival was 138.0 months (127.7–148.4), while in those with medium and high risk were 116.4 months (93.1–139.7), and 34.0 months (10.9–57.2) respectively (Log-rank (Mantel-Cox): *p* = 0.012) [Figure 3B]. The cumulative probability of tumor recurrence at 1 year, 3 years and 5 years in patients with low pre-MORAL risk was 0%, 3%, and 3%, respectively. For those with medium risk, the probabilities were 10%, 24%, and 24%, respectively. In patients with high risk, the probabilities were 33% at 1 year, 3 years, and 5 years respectively.

### 3.5. Mortality

The mean survival time in the overall cohort was 131.1 months (119.3–143.0), significantly lower in patients with HCC recurrence (56.4 months [22.7–90.0]) compared to those without recurrence (143.9 months [136.9–151.0]) (Log-rank (Mantel–Cox): *p* < 0.001) [Figure 2B]. The cumulative survival probability at 1, 3, and 5 years for patients with HCC recurrence was 77%, 32%, and 32%, respectively, compared to those without recurrence (98%, 96%, and 94%, respectively). Among the nine patients who experienced recurrence, six of them had died by the end of the study (66.7%), with HCC recurrence being the cause of death in all cases. The median survival time from the diagnosis of HCC recurrence to death was 13 months (IQR = 9–31.8).

## 4. Discussion

This retrospective observational study conducted in a tertiary hospital in northern Spain has revealed a 13.6% incidence rate of HCC recurrence, with 67% of cases occurring in extrahepatic locations. Notably, the majority of recurrences (75%) were observed within the first five years after transplantation. Factors such as BMI (obesity), vascular micro-invasion, and medium to high pre-MORAL risk were found to be associated with an elevated risk of recurrence, which subsequently correlated with a noteworthy rise in post-transplant mortality. 

Our study confirms the rates of post-liver transplantation HCC recurrence reported in previous studies, which range from 8% to 20% post-transplantation [5,9,17]. Moreover, it is well established that the most frequent sites of recurrence are the lungs, lymph nodes, and bones, which aligns with the recurrence pattern observed in our cohort [17]. In our series, HCC recurrence can be classified as early, with 56% recurring within the first three years. However, it is known that up to 75% of recurrences occur within the first two years after transplantation [3,5]. Although the difference may not be substantial, it could be attributed to factors such as the small sample size, inclusion of patients with favorable profiles based on Milan criteria and predominant use of bridging therapy, among others. Regarding mortality, by the end of the study, 66.7% of the patients who experienced recurrence had died, all due to causes related to tumor recurrence. The median survival time from the diagnosis of HCC recurrence to death was 13 months, consistent with previous literature [5,9].

Multiple risk factors for tumor recurrence have been described, primarily associated with tumor burden at diagnosis and explant, histological characteristics of the explant, tumor biology and treatment-related factors, as previously discussed. Combinations of these factors have resulted in the development of predictive indices or models, such as pre-Moral (including NLR, maximum AFP level, or largest tumor size), post-Moral (involving size and the number of nodules, vascular invasion and degree of differentiation in the explant), Combo-Moral (a composite score derived from both the pre-Moral and post-Moral scores) and the RETREAT model (a composite score calculated based on AFP level at LT, presence of microvascular invasion, and some of the largest viable tumor diameter plus the number of viable nodules) [12,13]. Our study results confirm the validity of these indices and prognostic markers. We identified two statistically significant factors: pre-Moral risk, with HR ranging from 20.4 (3.0–140.4) for medium risk to 30.2 (1.6–568.6) for high risk, and vascular micro-invasion, with HR of 8.8 (1.6–48.0). Microvascular invasion significantly influences the risk of tumor recurrence and survival, doubling the risk of death, and the prognostic value of the pre-Moral index has been previously discussed [5,12]. We were unable to confirm the association with other indices, such as post-Moral, Combo-Moral or RETREAT, either because they did not show statistical significance in the univariable analysis or because they were not included in the multivariable model to avoid collinearity and redundancies. The limited sample size, lack of high-risk cases in our series and the use of bridging therapy with consideration of only viable nodules in the explant may have influenced these results. Additionally, a noteworthy association was observed between increased recurrence risk and BMI with an HR of 1.3 (1.1–1.5). Indeed, when obesity was included in the model instead of BMI, it continued to show a significant association with the risk of TR in the multivariable analysis. The hazard ratio was 8.6 [(95% CI = 1.8–42.1); *p* = 0.008], while the other significant variables remained in the model. Despite a relatively weak association, our study provides support to the existing, albeit controversial, evidence linking obesity as a risk factor for the occurrence of hepatocellular carcinoma and its post-transplant recurrence [9,18,19,20,21]. This is especially relevant considering the increasing prevalence of metabolic-associated fatty liver disease (MAFLD) and its association with obesity [22,23]. Finally, the observed differences in immunosuppression therapy at the end of the first year in our study could be attributed to a tendency to transition to an mTOR inhibitor when identifying risk factors for TR post-transplantation.

The limitations of the study primarily stem from its retrospective and single-center nature, with a small number of patients, which likely resulted in limited power to estimate the obtained risks more accurately and identify other risk factors for HCC recurrence. The use of bridge therapy and the consideration of only viable tumors when quantifying the number and size of tumors in pre-transplant radiology and explant analysis may have limited the impact of these variables (and the models including them) on the estimation of TR risk. Finally, to prevent collinearity and redundancies, certain variables or scores were excluded from the multivariable analysis.

In conclusion, HCC recurrence after LT remains a significant challenge, and identifying risk factors and implementing preventive strategies is crucial. The results of our study emphasize the significance of meticulous patient selection, complemented by prognostic models as those mentioned here, along with appropriate surveillance and management strategies, to minimize the recurrence of HCC and enhance long-term outcomes after liver transplantation.

## Figures and Tables

**Table 1:** Characteristics of the study population.

Variable *	All (n = 66)	No Recurrence (n = 57)	Recurrence (n = 9)	*p*
***General***				
Sex (Male)	58 (87.9)	49 (86.0)	9 (100)	0.586
Age (years)	60 (53.5–63.5)	60.5 (57.0–64.0)	58.0 (53.5–62.5)	0.569
*BMI* (n = 65)	27.1 (25.9–28.2)	26.7 (25.6–27.8)	29.6 (25.3–33.9)	0.231
CV Risk factors				
Hypertension	22 (33.3)	17 (29.8)	5 (55.6)	0.147
Diabetes	20 (30.3)	16 (28.1)	4 (44.4)	0.437
Dyslipidemia	9 (13.6)	9 (15.8)	0 (0)	0.341
Smoker ^1^	50 (75.8)	42 (73.7)	8 (88.9)	0.436
Obesity ^2^ (n = 65)	13 (20.0)	9 (16.1)	4 (44.4)	**0.070**
Liver disease etiology ^3^				0.478
Alcohol (OH)	23 (34.8)	21 (36.8)	2 (22.2)	
Hepatitis C (HCV)	21 (31.8)	16 (28.1)	5 (55.6)	
Mixed (OH/HCV)	13 (19.7)	12 (21.1)	1 (11.1)	
MAFLD	2 (3.0)	2 (3.5)	0 (0)	
Autoim./Cholest.	2 (3.0)	2 (3.5)	0 (0.0)	
Others	5 (7.6)	4 (7.0)	1 (11.1)	
Neutroph. (×10^3^/mm^3^)	2.7 (2.0–4.3)	2.5 (1.9–4.2)	3.5 (2.7–5.1)	0.151
Lymphoc. (×10^3^/mm^3^)	1.2 (0.8–1.7)	1.2 (0.7–1.7)	1.1 (0.9–2.4)	>0.999
Platelets (×10^3^/mm^3^)	84 (63–115)	84 (66–114)	113 (58–136)	0.845
NLR	2.6 (1.7–3.7)	2.5 (1.6–3.7)	2.8 (1.7–5.1)	>0.999
PLR	83 (54–109)	85 (54.8–119.9)	75 (45.7–123.8)	0.473
*Pretransplant EGFR*	69.9 (68.7–71.2)	69.6 (68.4–70.8)	72.2 (67.1–77.4)	0.143
MELD	10 (8–12)	10 (8–12)	10 (8–11)	0.892
***Transplant***				
Waiting list time (mo.) (n = 63)	4.0 (2.0–8.0)	4.0 (1.8–8.0)	3.0 (2.0–11.5)	0.932
Time HCC-LT (mo.)	9.0 (5.0–15.5)	10.5 (5.0–17.3)	8.5 (4.5–12.0)	0.230
Donor age (y.) (n = 58)	65 (51.0–76.0)	65.0 (53.0–76.3)	52.0 (37.8–74.5)	0.743
Donor type				>0.999
Brain dead	61 (92.4)	52 (91.2)	9 (100.0)	
DCD	5 (7.6)	5 (8.8)	0 (0.0)	
Ischemia time (min.) (n = 62)	342 (263–420)	342 (266–428)	310 (221–414)	0.666
CMV status				
Low risk (D−/R−)	0 (0)	0 (0)	0 (0)	-
Med. risk (D±/R+)	61 (92.4)	53 (93.0)	8 (88.9)	0.531
Mismatch (D+/R−)	5 (7.6)	4 (7.0)	1 (11.1)	0.531
CMV infection ^4^	19 (28.8)	16 (28.1)	3 (33.3)	0.709
Histologic rejection	3 (4.5)	3 (5.3)	0 (0)	>0.999
Steroid bolus	5 (7.6)	5 (8.8)	0 (0)	>0.999
1st year immunosup ^3^				**0.029**
CNI monotherapy	45 (68.2)	39 (68.4)	6 (66.7)	
CNI + (MMF|EVR|P)	15 (22.7)	15 (26.3)	0 (0.0)	
EVR monotherapy	6 (9.1)	3 (5.3)	3 (33.3)	
***Pre-transplant tumor***				
AFP (waiting list incl.) (n = 64)	4.3 (2.4–11.9)	5.2 (2.4–12.0)	3.4 (2.6–12.2)	0.150
Pre-transplant AFP (n = 64)	4.2 (2.4–10.4)	4.5 (2.4–11.1)	3.4 (2.6–5.8)	0.150
*Nodules at dx.*	1.7 (1.5–1.9)	1.7 (1.4–1.9)	1.7 (1.0–2.3)	0.992
*Largest nod. dx.* (mm)	29.1 (26.7–31.6)	28.1 (25.5–30.7)	35.8 (28.6–42.9)	**0.028**
*Nodules at LT* ^5^	0.8 (0.6–1.1)	0.8 (0.6–1.1)	0.8 (-0.3–1.8)	0.410
*Largest nod. LT* ^5^ (mm)	11.6 (8.0–15.2)	12.2 (8.4–16.1)	7.7 (0.0–18.9)	0.225
HCC within Milan	65 (98.5)	56 (98.2)	9 (100.0)	>0.999
Confirmatory biopsy	7 (10.6)	6 (10.5)	1 (11.1)	>0.999
Downstage	0 (0)	0 (0)	0 (0)	
Bridging therapy ^3^				0.672
No	15 (22.7)	14 (24.6)	1 (11.1)	
RF/MW	37 (56.1)	34 (59.6)	3 (33.3)	
TACE	8 (12.1)	5 (8.8)	3 (33.3)	
Combined	6 (9.1)	4 (7.0)	2 (22.2)	
Pre-transpl. resection	9 (13.6)	9 (15.8)	0 (0)	0.341
***Tumor in liver explant***				
*Nodules* ^5^ *(number)*	0.7 (0.4–0.9)	0.6 (0.3–0.9)	1 (0.0–2.0)	0.463
*Nod. size sum* ^5^ (mm)	15.5 (9.4–21.7)	14.3 (7.8–20.7)	23.6 (0.8–46.3)	0.319
*Largest nodule* ^5^ (mm)	12.4 (7.9–16.7)	11.1 (6.9–15.49)	20.3 (0.2–40.4)	0.329
Microvasc. invasion (n = 65)	10 (15.4)	7 (12.3)	3 (37.5)	0.098
Differ. grade (n = 64) ^3^				>0.999
Well	21 (32.8)	17 (29.8)	4 (57.1)	
Moderate	13 (20.3)	13 (22.8)	0 (0.0)	
Poor	2 (3.1)	1 (1.8)	1 (14.3)	
Not available (necr.)	28 (43.8)	26 (45.6)	2 (28.6)	
***TR: Predictive models***				
NLR ⩾ 5	8 (12.1)	6 (10.5)	2 (22.2)	0.298
PLR ⩾ 125	12 (18.2)	10 (17.5)	2 (22.2)	0.663
pre-MORAL risk ^3^				**0.009**
Low	42 (63.6)	40 (70.2)	2 (22.2)	
Medium	21 (31.8)	15 (26.3)	6 (66.7)	
High	3 (4.5)	2 (3.5)	1 (11.1)	
Very high	0 (0)	0 (0)	0 (0)	
post-MORAL risk ^3^				0.287
Low	35 (53.0)	32 (56.1)	3 (33.3)	
Medium	30 (45.5)	25 (43.9)	5 (55.6)	
High	1 (1.5)	0 (0)	1 (11.1)	
Very high	0 (0)	0 (0)	0 (0)	
Combo-MORAL risk ^3^				0.077
Low	26 (39.4)	25 (43.9)	1 (11.1)	
Medium	30 (45.5)	26 (45.6)	4 (44.4)	
High	8 (12.1)	5 (8.8)	3 (33.3)	
Very high	2 (3.0)	1 (1.8)	1 (11.1)	
RETREAT risk ^3^				0.256
Low (0–3 points)	64 (97.0)	56 (98.2)	8 (88.9)	
Medium (4 points)	1 (1.5)	1 (1.8)	0 (0)	
High (⩾5 points)	1 (1.5)	0 (0)	1 (11.1)	
Months until TR	-	-	33.0 (13.5–60.0)	
***Mortality***				
Death from any cause	9 (13.6)	3 (5.3)	6 (66.7)	**<0.001**
Cause of death ^3^				**0.012**
HCC recurrence	6 (66.7)	0 (0)	6 (100)	
CV event	1 (11.1)	1 (33.3)	0 (0)	
De novo tumor	1 (11.1)	1 (33.3)	0 (0)	
Other causes	1 (11.1)	1 (33.3)	0 (0)	

Variables are classified into six main categories based on their relationships (e.g., General, transplant-related, etc.). Each category is prominently highlighted in bold and italics. Qualitative variables are expressed as the number of subjects and percentage (n; %), while quantitative variables are expressed as median and range (median; interquartile range) except for variables in italics which are expressed as mean and 95% confidence interval. ^1^ Former or active; ^2^ BMI ≥ 30; ^3^ To improve statistical robustness and meet the assumptions of the test, we grouped certain categorical variables to perform the Fisher’s exact test as follows: Liver disease etiology (alcohol vs. other etiologies), first-year immunosuppression (CNI in monotherapy or in combination versus EVR in monotherapy), bridging therapy (whether or not they received bridge therapy), differentiation grade (well-differentiated/necrotic tumor vs. moderate to undifferentiated), pre-MORAL, post-MORAL, and Combo-MORAL risks (low vs. higher grade), RETREAT risk (low vs. higher risk), and cause of death (HCC recurrence vs. others) ^4^ CMV infection requiring treatment (we did not have any cases of CMV disease); ^5^ Only viable tumors were considered in the analysis. * For variables with missing data, the number of patients is specified in parentheses. *p*-values < 0.05 have been highlighted in bold. Abbreviations: CV = Cardiovascular; BMI = Body Mass Index; MAFLD = Metabolic Associated Fatty Liver Disease; NLR = neutrophil-to-lymphocyte ratio; PLR = platelet-to-lymphocyte ratio; eGFR = Estimated Glomerular Filtration Rate based on creatinine using the CKD-EPI formula (expressed in mL/min/1.73 m^2^); MELD = Model for End-Stage Liver Disease; CMV = cytomegalovirus; HCC = hepatocellular carcinoma; CNI = calcineurin inhibitor; MMF = mycophenolate mofetil; EVR = everolimus; P = prednisone; RF = radiofrequency; MW = microwave; TACE = transarterial chemoembolization.

**Table 2:** Univariate and multivariate analysis of tumor recurrence risk using Cox regression.

	Univariable		Multivariable ^1^			
			Model 1 (BMI)		Model 2 (Obesity)	
Variables	HR (95% CI)	*p*	HR (95% CI)	*p*	HR (95% CI)	*p*
BMI	1.17 (1.01–1.36)	**0.037**	1.27 (1.07–1.51)	**0.006**	**-**	**-**
Obesity (ref. no)	3.99 (1.07–14.91)	**0.039**	-	-	8.62 (1.77–42.14)	**0.008**
Pre-transplant AFP	1.00 (0.99–1.02)	0.079				
Largest size at dx. (per mm of increment)	1.07 (1.00–1.13)	**0.036**				
Vascular microinvasion (ref. no)	4.83 (1.08–21.66)	**0.040**	8.77 (1.60–48.01)	**0.012**	8.49 (1.53–47.08)	**0.014**
Pre-Moral risk (ref. low)						
Medium risk	6.37 (1.28–31.72)	**0.024**	20.41 (2.97–140.38)	**0.002**	16.89 (2.49–114.82)	**0.004**
High risk	13.58 (1.18–185.89)	**0.036**	30.22 (1.61–568.64)	**0.023**	32.60 (1.52–701.34)	**0.026**
Post-Moral risk (ref. low)						
Medium risk	1.00 (0.26–3.79)	>0.999				
High risk	1.00 (0.00–high)	>0.999				
Combo-Moral risk (ref. low)						
Medium risk	3.96 (0.44–36.09)	0.221				
High risk	14.44 (1.37–152.42)	**0.026**				
Very high risk	35.54 (2.02–626.65)	**0.015**				
RETREAT risk (per point of increment)	1.67 (1.02–2.71)	**0.041**				

^1^ Two multivariable analysis models were constructed: Model 1 used BMI as a quantitative predictor variable, and Model 2 used obesity as a dichotomous predictor variable. To prevent redundancy and consider a more significant association found in the univariate analysis for pre-Moral and vascular micro-invasion, the multivariate analysis excluded the Post-Moral, Combo-Moral and RETREAT scores, as well as the largest size at diagnosis. *p*-values < 0.05 have been highlighted in bold.

## Data Availability

The data presented in this study are available upon request from the corresponding author, provided a justified request is made. The data are not publicly available due to confidentiality and data protection concerns.

## Abbreviations

LT	Liver transplantation
HCC	Hepatocellular carcinoma
TR	Tumor recurrence
MORAL	Model of Recurrence After Liver Transplantation
BMI	Body Mass Index
AFP	alpha-fetoprotein
NLR	neutrophil to lymphocyte ratio
RETREAT	Risk Estimation of Tumor Recurrence After Transplant
MELD	Model for End-Stage Liver Disease
MELD-Na	Model for End-Stage Liver Disease-sodium
HIV	Human immunodeficiency virus
CMV	Cytomegalovirus
IQR	Interquartile range
CNI	calcineurin inhibitors
mTOR	mammalian target of rapamycin
